# Redox Roles of Reactive Oxygen Species in Cardiovascular Diseases

**DOI:** 10.3390/ijms161126059

**Published:** 2015-11-20

**Authors:** Feng He, Li Zuo

**Affiliations:** 1 Department of Kinesiology, California State University-Chico, Chico, CA 95929, USA; fhe@csuchico.edu; 2 Molecular Physiology and Rehabilitation Research Lab, Radiologic Sciences and Respiratory Therapy Division, School of Health and Rehabilitation Sciences, the Ohio State University College of Medicine, Columbus, OH 43210, USA; 3 Interdisciplinary Biophysics Graduate Program, the Ohio State University, Columbus, OH 43210, USA

**Keywords:** free radical, reactive oxygen species, atherosclerosis, antioxidant

## Abstract

Cardiovascular disease (CVD), a major cause of mortality in the world, has been extensively studied over the past decade. However, the exact mechanism underlying its pathogenesis has not been fully elucidated. Reactive oxygen species (ROS) play a pivotal role in the progression of CVD. Particularly, ROS are commonly engaged in developing typical characteristics of atherosclerosis, one of the dominant CVDs. This review will discuss the involvement of ROS in atherosclerosis, specifically their effect on inflammation, disturbed blood flow and arterial wall remodeling. Pharmacological interventions target ROS in order to alleviate oxidative stress and CVD symptoms, yet results are varied due to the paradoxical role of ROS in CVD. Lack of effectiveness in clinical trials suggests that understanding the exact role of ROS in the pathophysiology of CVD and developing novel treatments, such as antioxidant gene therapy and nanotechnology-related antioxidant delivery, could provide a therapeutic advance in treating CVDs. While genetic therapies focusing on specific antioxidant expression seem promising in CVD treatments, multiple technological challenges exist precluding its immediate clinical applications.

## 1. Introduction

Cardiovascular disease (CVD) (e.g., peripheral vascular disease, coronary heart disease, stroke, and myocardial infarction) is not only the leading cause of mortality in United States, but also a rising cause of death in both undeveloped and developed countries [1]. According to the American Heart Association in 2015, the prevalence of total coronary heart disease is 6.2% in adults (age ≥ 20) and the direct and indirect financial costs of all different cardiovascular diseases surged to $320.1 billion in United States in 2011 [2]. In particular, atherosclerosis is the dominant cause of more progressive CVD and is characterized as an accumulation of inflammatory cells in the arterial wall due to a variety of trauma to the endothelial and smooth muscle cells. Thus, atherosclerotic plaques are formed leading to the increased arterial thickness and stiffness [3,4]. The chronic progress of inflammatory plaques can narrow blood vessels and potentially block blood flow, causing a malfunction of the local tissue due to lack of oxygen and nutrients. The rupture of the vulnerable plaque can form a thrombus which may occlude the coronary and/or cerebral blood circulation leading to myocardial infarction and stroke [1,5].

Obesity, cigarette smoking, hyperlipidemia, hyperglycemia, and a sedentary lifestyle are typical risk factors associated with CVD [6,7]. Particularly, endothelial dysfunction is linked with high-risk populations [8]. Of note, those who have a family history of heart disease are more likely to develop CVD at a later stage of their life [9]. Although the pathophysiology of atherosclerosis has not been fully elucidated, excessive ROS production plays an essential role in the development of CVD [8]. The increase in ROS production derived from dysfunctional endothelial and vascular smooth muscle cells contributes to a series of vascular restructures (e.g., the proliferation of vascular smooth cells, thickening of the intima, remodeling of the artery, pulmonary hypertension and changing in fluid dynamics), leading to blood vessel stenosis and atherosclerosis [10,11,12].

Numerous antioxidant interventions have been investigated in cell cultures, animal studies, and clinical trials to examine the redox-regulated mechanisms underlying the pathophysiological development of CVD [13,14,15,16]. A more thorough understanding of ROS in the pathophysiology of CVD could lead to new antioxidant-associated therapies in the treatment of the disease.

## 2. Potential Sources of ROS (Reactive Oxygen Species)

Oxidative stress normally occurs when the balance between ROS production and antioxidant defense capacity is disrupted in favor of the former [17,18]. ROS include superoxide (O_2_^•−^), hydroxyl radical (^•^OH), hydrogen peroxide (H_2_O_2_), and peroxynitrite (ONOO^−^) [19,20]. Wattanapitayakul *et al.* [21] suggested that ROS production is resulted from the uncoupled mitochondrial electron transport chain, thus, ROS can be a by-product of normal metabolism [22]. For example, about 1%–3% of oxygen is converted to O_2_^•−^ via oxidative phosphorylation in mitochondrial complexes I and III [23,24]. It is well known that cardiac myocytes have a high density of mitochondria. Therefore, the consumption of oxygen in cardiac myocytes is relatively higher which potentially predisposes the cells to oxidative stress [25]. Other intracellular antioxidants including superoxide dismutase (SOD), glutathione peroxidase (GPx), catalase and other non-enzyme antioxidants (e.g., vitamin C, vitamin E, β-carotene, bilirubin, *etc.*) keep ROS at homeostatic levels [22]. Although ROS are essential in cell signaling pathways, excessive ROS production leads to damage of the cell membrane, proteins, and DNA, contributing to striated muscle fatigue or dysfunction [26,27,28,29]. Inflammation-related ROS formation can be increased during an innate defensive response. Xanthine oxidase (XO) contributes to increased O_2_^•−^ generation, which can be blocked by the XO inhibitor, such as allopurinol, to improve heart conditions [25]. NADH/NADPH oxidase (Nox), generally located in the plasma membrane of the cells, is activated during phagocytosis, contributing to ROS production [30,31]. The upregulation of Nox2 and Nox4 is associated with the elevated oxidative stress seen during CVD [32,33,34]. For instance, Kuroda *et al.* [32] found that Nox4^−/−^ mice showed augmented cardiac hypertrophy whereas Nox4 overexpression worsened the cardiac function. Thus, Nox is another main contributor of oxidative stress in the mitochondrial redox systems [32]. Nevertheless, the exact redox mechanism in CVD is yet to be completely understood. Vendrov *et al.* [35] suggested that mitochondrial oxidative stress and Nox4 activity are pivotal mediators in age-related CVD. De Marchi *et al.* [36] reviewed the crucial roles of protein kinase C and an isoform of growth factor adaptor (p66^Shc^) in CVD and other metabolic diseases, such as obesity and diabetes, through regulation of redox pathways.

## 3. ROS in Ischemia-Reperfusion Damage

In 1989, Bolli *et al.* [37] provided direct evidence that ROS were dramatically increased in the intact canine myocardium following ischemic occlusion using electron paramagnetic resonance. This result was further confirmed in a recent study by Zhu *et al.* [38]. Reperfusion of coronary blood flow back to the ischemic myocardium can paradoxically evoke further damage to the microvascular function, resulting in arrhythmias [39]. Nitroglycerin, a common prescription drug for patients with myocardial ischemia and congestive heart failure, was reported to have a protective effect on the endothelium against ischemia-reperfusion injuries [40]. Yet the protective effect of long-term administration of nitroglycerin on CVD remains unknown [41]. Additionally, the increase in ROS release as well as opening of the mitochondrial permeability transition pore may play a mediating role in the protection against ischemia-reperfusion damage in endothelium [40]. This is also the potential mechanism involved in ischemic preconditioning (IPC), which was first introduced by Murry *et al.* [40,42,43] that demonstrates a strong protective effect against ischemia injuries in myocardium. Dogs subjected to short periods of ischemic conditions prior to a 40-min occlusion had smaller infarct size than the controls without the short exposure to ischemia. However, the molecular pathway of this protective effect was not fully understood at that time [43]. Lebuffe *et al.* [44] revealed that ROS and nitric oxide (NO) regulate downstream pathways that are required for this protective effect by activating mitochondrial K_ATP_ channels. Interestingly, Crisafulli *et al.* [41] in 2004 first reported that exercise-induced myocardial preconditioning (e.g., the improvement of hemodynamics) has a similar effect with widely used pharmaceutical therapeutic tools (e.g., transdermal administration of nitroglycerin) in patients with stable angina.

## 4. ROS and Atherosclerosis

Risk factors such as obesity, aging, and hypertension are all associated with the occurrence of atherosclerosis [6]. Aging is linked to the biochemical and morphological remodeling of the artery which contributes to the incidence of heart failure or stroke [45,46]. Li *et al.* [47] confirmed the presence of vascular senescence (*i.e.*, thickened intima) and the molecular modifications (*i.e.*, the increase in matrix metalloproteinase-2, transforming growth factor-β, intracellular adhesive molecule-1, and fibronectin protein levels) in aortas of the aged rats. One of the human adhesion molecules, vascular cell adhesion molecule-1 (VCAM-1), which facilitates the adherence of mononuclear cells to endothelium, was upregulated upon cardiac risk factors such as hypertension and diabetes. Therefore, this study suggests that VCAM-1 plays a role in the pathogenesis of atherosclerosis [10]. VCAM-1 was expressed in the areas that are predisposed to the development of atherosclerosis in animal models [48]. Overall, atherosclerosis has three quintessential characteristics: 1. Inflammation; 2. Disturbed blood blow and abnormal shear stress; and 3. Arterial wall remodeling. Excessive ROS production plays a role in all these features [10]. Patel *et al.* [49] and Park *et al.* [50] confirmed that ROS contribute to the structural remodeling through smooth muscle proliferation and enhanced inflammation.

Fluid shear stress, as a mechanic force against vascular endothelium due to blood flow, is a crucial factor in the development of atherosclerosis. The changes in blood flow patterns including disturbed oscillatory shear stress, low flow, and turbulent fluid could adversely impact endothelial function. The low, reverse, or oscillated shear stress in arteries has pro-inflammatory and pro-oxidative effects on blood vessels whereas laminar flow has an antioxidant and anti-inflammatory role [51,52,53]. The distribution of the disturbed non-laminar flow is not randomly determined. It commonly occurs at branch points, bifurcations, and curvatures [54]. The study by Hwang *et al.* [55] suggested that the chronic exposure to non-laminar shear stress of arterial regions stimulates O_2_^•−^ generation induced by endothelial Nox, leading to monocyte adhesion. The upregulation of adhesion molecules including P-selectin, VCAM-1, and E-selectin induces further inflammation by attracting more leukocytes. The increased inflammatory response exacerbates ROS production via phagocytosis, which is a critical factor in the early stage of atherosclerosis [55,56].

It is well established that Nox is a major source of ROS in the vascular system. Nox has been expressed not only in phagocytic cells (e.g., neutrophils and macrophages), but also in endothelial cells, smooth muscle cells and fibroblasts [57]. Five components of Nox have been identified (p22^phox^, p47^phox^, p67^phox^, gp91^phox^, and rac) in endothelial cells [58]. Nevertheless, these components do not contribute equally to shear stress-induced O_2_^•−^ production. Hwang *et al.* [55] revealed that p47^phox^ is the major contributor for the ROS production induced by oscillatory shear stress which sheds light on the development of effective antioxidant interventions.

During the inflammatory stage of atherosclerosis, sources of ROS may include the infiltrated monocytes/macrophages, dysfunctional endothelial cells, and smooth muscle cells that migrated from media to intima [56,59]. The damage to the atrial wall was initiated by the transmigration of oxidized low-density lipoprotein (LDL) from blood vessel lumen to the tunica media [60]. LDL that entered the arterial wall was oxidized by excessive ROS and scavenged by macrophages, forming lipid drops, which are characterized as foam cells [61]. This is a one of the critical steps in the development of atherosclerosis [25]. Furthermore, matrix metalloproteinase secreted from endothelial cells, foam cells, and vascular smooth cells is activated during oxidative stress in response to inflammation and non-laminar shear stress, which in turn degrades the extracellular matrix components that build up in the arterial wall leading to the ruptures of thrombosis [51].

## 5. Pharmacological Interventions Targeting ROS Sources in CVD (Cardiovascular Disease)

Current drugs for vascular protection include statins as well as angiotensin-converting enzyme (ACE) inhibitors. Although statin, a popular blood cholesterol reducer, does not directly scavenge ROS, it acts as an indirect antioxidant to inhibit the 3-hydroxy-3-methyl-glutaryl-coenzyme A (HMG CoA) reductase pathway. HMG CoA reductase contributes to the production of farnesyl pyrophosphate and geranylgeranyl pyrophosphate, the critical steps for O_2_^•−^ generation from Nox. The efficacy of statins, the widely prescribed HMG CoA reductase inhibitors in reducing the incidence of cardiac events and mortality, is likely increased by their potential antioxidant properties and their ability to enhance endothelial nitric oxide synthase (eNOS) expression [62,63,64,65]. Statins can reverse the downregulation of eNOS expression induced by hypoxia and oxidized LDL, underlying their capacity to improve the vascular NO bioactivity and plaque stability independent of their lipid-lowering effects [66,67,68]. Statins also prevent the downregulation of eNOS induced by tumor necrosis factor [69]. These effects may play a critical role in the development of chronic statin therapy for the primary and secondary prevention of coronary heart disease. In addition, a variety of antioxidants such as vitamin C and E, beta carotene, quercetin, lycopene, and CoQ10, have been investigated in terms of their preventative and/or therapeutic effects on atherosclerosis, ventricular remodeling, ischemia-reperfusion heart injury, heart failure and myocardial infarction [10,70,71,72,73].

ACE can convert angiotensin I to angiotensin II. Increased angiotensin II activity is essential for arterial wall remodeling, such as shrinking of the lumen diameter and thickening of the media, which are key characteristics of atherosclerosis [74,75]. Specifically, high angiotensin II levels have been shown to contribute to the release of vascular O_2_^•−^ [76]. Muller *et al.* [77] observed that NO exhibited antioxidative function by decreasing O_2_^•−^ bioavailability in the thoracic aorta of a hypercholesterolemia rabbit model. However, O_2_^•−^ can react with NO to lower its concentration, thereby exacerbating the progression of atherosclerosis [78]. Angiotensin II type I receptor blocker (ARB) enhances the plasma levels of angiotensin II to stimulate angiotensin II type II receptors, and subsequently lead to NO production and vasodilation [79,80]. In the clinical trial performed by Ono *et al.* [80] hypertensive patients treated with candesartan (a type of ARB) exhibited markedly decreased carotid intima-media thickness, largely via elevated NO production and lowered oxidative stress. Furthermore, the combination of ARB and ACE inhibitor (ACEI) produces an additive inhibitory effect on oxidative stress in a rat model of carotid artery injury [81].

It is also worth noting that calcium channel blockers (CCB) (e.g., amlodipine, lacidipine, and nisoldipine) are widely used for CVD treatment due to their antihypertensive and antioxidant effects [82]. In a meta-analysis of 27 clinical trials, Costanzo *et al.* [83] investigated the effect of CCB on patients with CVD, concluding that CCB reduce the risk of CVD and stroke compared with traditional treatment, ACEI. Nevertheless, the potential beneficial effect of CCB (e.g., antiatherogenic, the inhibition of vascular proteoglycan synthesis), other than antihypertensive actions, do not further attenuate the progression of atherosclerosis [84]. Nicotinic acid (niacin) is also a commonly used treatment for CVD due to its favorable action in reducing LDL and increasing high-density lipoprotein. In addition, niacin was shown to inhibit vascular inflammation, angiotensin II-induced ROS generation, and LDL oxidation in atherogenesis. Thus, further investigation is necessary to explore this new role of niacin in the treatment of CVDs [85,86].

Collectively, pharmaceutical approaches for the treatment of CVD seem to have limited efficiency. Studies exploring the association between inflammation, NO, and oxidative stress may provide insights into the development of novel therapeutics. The integration of multiple prevention methods through nutrition, nutraceuticals using vitamin B6, vitamin C, and coenzyme Q10, stress management, and exercise may promote health and vitality [87]. Alexander *et al.* [87] summarized that both nutrition and nutraceuticals were capable of mimicking antihypertensive medications (e.g., diuretics, beta-blockers, and direct vasodilators). Houston *et al.* [88] found that 62% of hypertensive patients were able to discontinue their antihypertensive drugs following a six-month natural intervention with nutraceuticals and healthy lifestyle modifications.

ROS play an essential role in the initiation, development, and progression stages of CVD, and are linked to other CVD risk factors such as diabetes and hypertension [6,89,90]. Thus, it is important to develop strategies to minimize excessive ROS production and/or boost antioxidant defense systems to maintain redox balance. Lane *et al.* [91] conducted a cross-sectional study by using the National Health and Nutrition Examination Survey. They reported that specific dietary supplements, such as vitamin A, C and E, were associated with lower incidence of peripheral arterial disease, irrespective of other traditional risk factors [91]. Back in 1996, Stephens *et al.* [92] conducted a double-blind, placebo-controlled study in 2002 on patients with coronary disease. They found that vitamin E treatment (400 or 800 IU per day) significantly reduced the rate of non-fatal myocardial infarction of the patients. Recently, Ashor *et al.* [93] performed a systemic review and meta-analysis to investigate the effects of antioxidant vitamins on atrial stiffness by measuring post-wave velocity and augmentation index based on 20 randomized controlled trials. They concluded that antioxidant vitamins had a slight protective effect by alleviating arterial stiffness in adults and the effectiveness was dependent on supplementation dose and duration. Those participants who had lower baseline levels of circulating vitamin E have achieved a better therapeutic effect from interventions.

Despite these positive results, the effectiveness of antioxidant intervention in clinical trials for CVD therapy remains controversial. Miller *et al.* [94] performed a meta-analysis of 19 clinical trials, and concluded that high-dose (≥400 IU per day) of vitamin E potentially increases mortality. Therefore, the type, dose, and duration of the antioxidant supplementation as well as the variety of the population subjected to the studies might contribute to the mixed results [1,36].

It is well established that ROS play a dual role in both the pathophysiology and physiology of the cardiovascular system. Moderate ROS production has a protective effect in IPC strategies for patients with myocardium ischemia and heart failure [42,95,96]. However, excessive ROS generation can disturb cellular signaling pathways and result in cellular damage worsening the development of CVD [97]. It is critical to identify the location of the disrupted redox balance and excessive concentrations of ROS in the cardiovascular system for the prevention and treatment of CVD. Furthermore, matching antioxidant therapies to the characteristics, stage, concentration, and location of the oxidative stress at the optimum time is not only indispensable for securing the efficiency of the treatment, but is also extremely challenging for future therapeutic approaches.

## 6. Technological Advance and Challenges in Genetic Antioxidant Therapies in CVD

Nuclear E2-related factor-2 (Nrf2) has been intensively investigated due to its important function as a major regulator of the expression of multiple critical antioxidant and detoxification genes [98]. When organisms are subjected to oxidative stress or other stresses, Nrf2, a transcription factor, translocates into the nucleus, binding to the antioxidant response element (ARE), which, in turn, regulates the gene expression of numerous antioxidant-related enzymes, such as heme oxygenase-1 (HO-1) and enzymes associated with glutathione metabolism [98,99]. *Nrf2* gene transfer or application of Nrf2-inducing drugs suppressed smooth muscle cell proliferation via HO-1 mediation *in vitro* and decreased oxidative stress by inhibiting inflammatory response in rabbit aortas [99]. This is the first *in vivo* study to demonstrate that *Nrf2* gene transfer reduces oxidative stress and inflammation in the blood vessel wall. Bräsen *et al.* [100] examined the local gene therapy with extracellular superoxide dismutase (EC-SOD) on stent-induced vascular injury in rabbit aortas and found that the EC-SOD gene therapy facilitates endothelial recovery in atherosclerotic vessels. Furthermore, HO-1 catalyzes the heme degradation and yields bilirubin, iron and carbon monoxide [101]. Otterbein *et al.* [102] revealed that HO-1 can serve as a “therapeutic funnel” by its anti-apoptotic, anti-inflammatory, and anti-proliferative roles. The end-products of HO-1 catabolization play an important protective role during ischemia-reperfusion injury and intimal proliferation following balloon injury in rodent studies.

Mounting *in vitro* and *in vivo* evidence has shown that excessive oxidative stress is responsible for the pathophysiological development of CVD. Thus, antioxidant supplementation that is able to scavenge O_2_^•−^ or convert free radicals to less active species could potentially improve CVD. However, lack of efficient therapeutic effects from various antioxidant interventions in clinical trials suggest that establishing biomarkers of oxidative stress and developing more stable antioxidant and delivery methods could facilitate the translation from bench to bedside in CVD. Accordingly, Jain *et al.* [103] mentioned that novel antioxidant delivery strategies, such as using nano-formulated particle carrier or liposomes to target specific organ and tissue, can provide a therapeutic promise for the treatment of CVD. Future perspectives in antioxidant gene therapy include: (1) Genetically engineering cells by using retroviral *ex vivo* gene transfer of progenitor or stem cells; (2) Therapies that focus on transcription factors that integrate the responses of multiple antioxidant and/or anti-inflammatory genes (e.g., Nrf2, hypoxia-inducible factor 1-α); and (3) Novel gene delivery methods [99].

## 7. Summary

The exact redox mechanism of CVD is complex and has not yet been fully determined. ROS play a significant role in the pathogenesis and development of CVD, chiefly as an instigator of atherosclerosis. While we have yet to qualitatively describe the complete mechanism in CVD, ROS redox therapy shows signs of being a probable treatment option. Future studies will seek to investigate the possibility of genetically modifying antioxidant expression as a novel therapeutic option for CVD.
